# Postcarotid Endarterectomy Hematoma Induced Arrhythmia: Report of a Rare Case

**DOI:** 10.1155/2023/4633731

**Published:** 2023-12-04

**Authors:** George Galyfos, Alexandros Chamzin, Fragiska Sigala, Konstantinos Filis

**Affiliations:** Vascular Unit, First Department of Propedeutic Surgery, National and Kapodistrian University of Athens, Athens, Greece

## Abstract

Postcarotid endarterectomy (CEA) hematomas are common although they are rarely threatening and necessitate reoperation. We aim to report a rare case of an expanding hematoma that caused a cardiac arrhythmia (bigeminy) which was reversed after hematoma evacuation.

## 1. Introduction

Cervical hematomas can be a severe complication after carotid endarterectomy (CEA), necessitating even reoperation in some cases. However, the overall incidence of postoperative hematomas needing evacuation remains low [1]. If the hematoma is large or expanding, it can lead to airway obstruction and it can even be life-threatening [2].

Additionally, major cardiac events such as myocardial infarction (MI), cardiac arrhythmias, or heart failure have been reported in the early setting after carotid surgery. Overall, the incidence of major cardiac events post-CEA ranges from 2 to 4% in the literature [3–5]. Although most of the cardiac events are benign, they have been associated with long-term mortality necessitating a closer follow-up in such patients [4, 5].

We aim to report a rare case of a post-CEA hematoma that caused a bigeminy arrhythmia and needed evacuation. We further discuss on proper management.

## 2. Case Report

**Figure 1:**
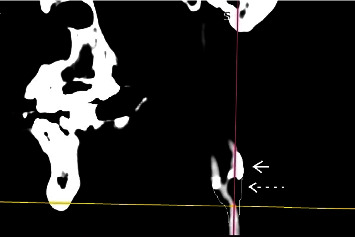
Computed angiography showing a significant internal carotid artery stenosis, with intraplaque thrombus (white arrow, calcification; interrupted arrow/black area, intraplaque thrombus).

A 57-year-old male patient presented with recent left-sided amaurosis fugax. His medical history included BMI > 30 kg/m^2^, heavy smoking (60 pack-years), dyslipidemia, resistant arterial hypertension (160/70 mmHg under triple regimen including beta-blocker, Ca channel blocker, and angiotensin converting enzyme (ACE) inhibitor), coronary artery disease (myocardial infarction and angioplasty plus 2 stents less 1 month ago, under dual antiplatelet treatment (ASA 100 mg plus clopidogrel 75 mg)), and renal dysfunction (creatinine = 2.2 mg/dl). Ultrasound and computed angiography (CTA) revealed a left 70% internal carotid artery (ICA) stenosis with intraplaque thrombus. CTA did not reveal any typical ischemic lesion in the brain nor any significant deficiency in the brain circulation (Figure 1).

**Figure 2:**
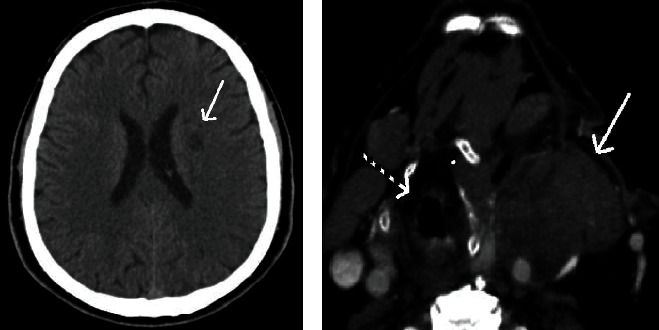
Computed tomography showing (a) a small ischemic lesion in the left white matter (arrow) and (b) a large cervical hematoma (white arrow) with deviation of the trachea (interrupted arrow).

The patient underwent CEA with synthetic patch placement. Due to anatomical difficulties (thick and short neck, high carotid bifurcation in CTA), placement of a shunt was not feasible although ICA backflow was unsatisfying (stump pressure measured: 39 mmHg). Although anatomical difficulties were suspected preoperatively, CEA was preferred over stenting as this was a symptomatic case. Carotid clamping time was 22 minutes. During awaking from anesthesia, the patient presented confusion and right-sided hemiparesis and a very high systolic arterial pressure (>180 mmHg). Under conservative treatment including IV antihypertensives, the patient improved within the next hours. Brain imaging revealed a small infarct in the left white matter (possibly embolic) with possible oedema as well as a large cervical hematoma with trachea deviation (Figure 2). No heart arrhythmia was evident at that time. However, the patient complained only for light difficulty in swallowing without any symptoms related to the airway. Due to comorbidities, a conservative treatment of the hematoma was decided initially. The patient was discharged after 5 days, with normal neurologic status, regulated hypertension, normal heart rhythm, and a stable cervical hematoma (without swallowing or breathing difficulty). The patient was discharged under ASA only for a few days due to the hematoma after consulting with the cardiologist.

**Figure 3:**
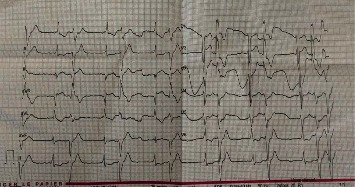
Electrocardiogram showing the bigeminy arrhythmia (1 : 1 ratio extrasystole/sinus-triggered QRS).

A week after, the patient presented with an enlarged hematoma and a bigeminy heart rhythm (asymptomatic arrhythmia, Figure 3). Laboratory testing indicated no abnormal levels of electrolytes such as calcium, magnesium, potassium, and phosphate. The patient underwent an urgent reoperation including hematoma excision, hemostasis, and wound revision. The heart rhythm recovered to normal sinus rhythm right after the hematoma removal. The patient was discharged after 3 days with normal neurologic status and under antibiotics and dual antiplatelet treatment (due to the recent cardiac stenting). The further wound course was uneventful. The arterial pressure was better regulated, and the patient is under nephrologist consultation. Three months after surgery, the patient remains asymptomatic.

## 3. Discussion

Post-CEA hematomas are more frequently observed compared to other adverse events such as cranial nerve injury, respiratory complications, or major cardiac events [6]. Overall, postoperative hematoma incidence can reach up to 25% among studies [7]. A potentially avoidable complication, cervical hematomas increase the risk of infection, wound breakdown, fistulae, and tracheal or oesophageal obstruction [6]. Additionally, patients who develop a neck hematoma have a significantly higher rate of mortality, operative stroke, and MI and require more blood transfusions and longer hospital stays [8]. However, the rate of hematomas necessitating reintervention is much lower ranging from 4% to 12% [7]. In a large study, Morales Gisbert et al. reported that almost two-thirds of the bleeding cases necessitated a reoperation [9]. Symptoms may vary and be nonspecific. In more than half of the patients, only a neck swelling is present without any symptoms affecting the airway or without any significant haemoglobin decrease. This concurs with our case where the patient initially presented with nonspecific symptoms and without any threat to the airway.

In most cases, threatening neck hematomas form within the first 24-hour postsurgery, and reoperation occurs within 6 hours from the original procedure if necessary [2]. This does not concur with our case, where the increase of the hematoma was slower and evacuation was performed more than one week after the initial procedure. Various factors have been associated with higher risk for hematoma occurrence such as comorbidities, blood disorders, anticoagulant treatment, preoperative platelet levels, surgical technique, use of shunt, protamine infusion, and poor hypertension control [7, 10, 11]. Ferreira et al. have found that previous clopidogrel use, especially if it is used 24 h before surgery, is independently associated with bleeding and reoperation [7]. This concurs with our case where the patient was under dual antiplatelet therapy. The majority of hematomas in such cases (almost 80%) result from capillary oozing whereas only 20% result from arteriotomy defects [1]. Therefore, meticulous surgical technique in obtaining hemostasis, control of postoperative hypertension, and wound drainage when indicated will help reduce the incidence of postoperative wound hematoma. Finally, the use of direct pressure following CEA in the immediate postoperative period has shown to significantly reduce the risk for hematoma formation [6].

One important issue that arises is the proper antithrombotic treatment among patients undergoing CEA. Current guidelines recommend that an antiplatelet monotherapy should be prescribed for patients undergoing CEA [12]. Clopidogrel is preferred for patients who were symptomatic before surgery although either clopidogrel or ASA could be prescribed for asymptomatic patients [12, 13]. In general, dual antiplatelet is not recommended for postoperative therapy unless there is a cardiac or other indication such as in our case [12]. This is to reduce any risk for bleeding associated with unnecessary combined therapy. However, dual antiplatelet therapy cannot be easily stopped in cases with recent coronary interventions due to the high risk for coronary stent thrombosis, such as in our case [13]. Finally, recent data indicate that even for high-risk patients such as patients with recent MI, CEA seem to be associated with lower stroke/death/MI risk compared to stenting [14].

The majority of major cardiac events post-CEA occur within the first 7 days from surgery [3]. Arrhythmias seem to occur more frequently compared to other cardiac events such as MI or congestive heart failure [3]. The relative increase in sympathetic modulation after CEA seems to be mediated by alterations in the sensitivity of carotid sinus baroreceptors [15]. However, 75% of those events seem to be minor (such as paroxysmal ventricular contraction and supraventricular tachycardia), occur temporarily, and could be controlled with medications and supportive treatments [3, 16]. On the other hand, studies have shown that reduction of the vagal nerve tone can also lead to heart arrhythmias [17]. This could be activated through pressure applied on the nerve by masses such as a tumour or a large hematoma. Additionally, pressure applied on carotid sinus itself has been associated with heart rhythm abnormalities such as ventricular fibrillation or bradycardia [18]. One could argue that the surgical stress or the postoperative ischemic cerebral event could have initiated the arrhythmia in our case. However, the arrhythmia presented a week after the operation and after the patient's neurological status was normalized. Additionally, both the occurrence of the bigeminy after the enlargement of the hematoma and the heart rate recovery after its excision reveal a possible causative relation.

Only one case has been reported of a cervical mass associated with heart arrhythmia that was relieved after mass resection [19]. In that case, however, the mass was a cervical vagal schwannoma complicated with hematoma. It could be argued that the hematoma applied pressure against the vagus and that could have some effect on the heart rhythm. It has been reported that the left vagal nerve has a greater influence on atrioventricular conduction, concurring with our case [19, 20]. Intraoperative manipulation of the vagus nerve tends to induce cardiac and circulatory fluctuations in patients, resulting in bradycardia and reduced blood pressure [20, 21]. Pressure on the vagus nerve can cause reduction of its parasympathetic tone and loss of the protective synergistic effect on the heart function. This could explain the cause of the arrhythmia as well as why the arrhythmia was reversed after the hematoma evacuation in our case.

In conclusion, conducting CEA in patients with anatomical difficulties and high bleeding risk is always a challenge increasing the risk for postoperative hematomas. Increasing neck hematomas could possibly lead to heart rate abnormalities through pressure appliance on the vagus or the carotid sinus. Therefore, early hematoma evacuation is necessary when threatening the airway, expanding or causing other complications such as arrhythmias.
